# An emerging probiotic with liver health benefits for smokers

**DOI:** 10.1093/lifemeta/loac033

**Published:** 2022-11-17

**Authors:** Wei Jia, Xiaojiao Zheng

**Affiliations:** Center for Translational Medicine and Shanghai Key Laboratory of Diabetes Mellitus, Shanghai Sixth People’s Hospital Affiliated to Shanghai Jiao Tong University School of Medicine, Shanghai 200233, China; School of Chinese Medicine, Hong Kong Baptist University, Kowloon Tong, Hong Kong 999077, China; Center for Translational Medicine and Shanghai Key Laboratory of Diabetes Mellitus, Shanghai Sixth People’s Hospital Affiliated to Shanghai Jiao Tong University School of Medicine, Shanghai 200233, China

## Abstract

Cigarette smoking is considered a risk factor for nonalcoholic fatty liver disease (NAFLD). In a study recently published in *Nature*, Chen *et al.* unveiled a mechanistic role of nicotine in NAFLD progression. In addition, they identified a gut bacterium *Bacteroides xylanisolvens* that can reduce intestinal nicotine levels, and thus improve nicotine-induced NAFLD phenotypes in mice.

Tobacco use is a leading cause of preventable disease and death worldwide. The number of smokers worldwide has been reported to be over 1.1 billion, accounting for about 15% of the global population [1]. Although China has made great efforts in tobacco control in the last two decades, there are over 300 million smokers and an even greater number of passive smokers nationwide [2]. Clinical studies have shown that smoking is strongly associated with the development of nonalcoholic fatty liver disease (NAFLD) [2–5], but the mechanism remains unclear. NAFLD is a major metabolic disease that may further progress to nonalcoholic steatohepatitis (NASH), which is mainly manifested by accumulation of lipids in the liver, an inflammatory process, and fibrosis which may further progress to irreversible cirrhosis or even liver cancer. The causative factors and pathological mechanisms of NASH are not well understood, and to date, there have been no FDA-approved therapeutic drugs for NASH.

Gut microbiota have coevolved with humans and interact with host organs through multiple pathways that are involved in a number of metabolic processes of the body throughout the life cycle [6, 7]. Previous studies by Jiang *et al*. have demonstrated that metabolites derived from host−gut microbiota cometabolism, such as ceramides, play a key role in the onset and development of NAFLD [8]. In the current study, the same research team led by Jiang reported their interesting findings regarding how smoking promotes NAFLD [9]. They revealed that nicotine accumulates in the intestine during smoking and activates the intestinal epithelial AMPKα-SMPD3-ceramide axis to promote NASH development through the intestine–liver crosstalk. More interestingly, they identified a gut bacterial species, *Bacteroides xylanisolvens* (*B. xylanisolvens*), which can effectively degrade intestinal nicotine, alleviating the progression of smoking-related NASH (Fig. 1).

**Figure 1:**
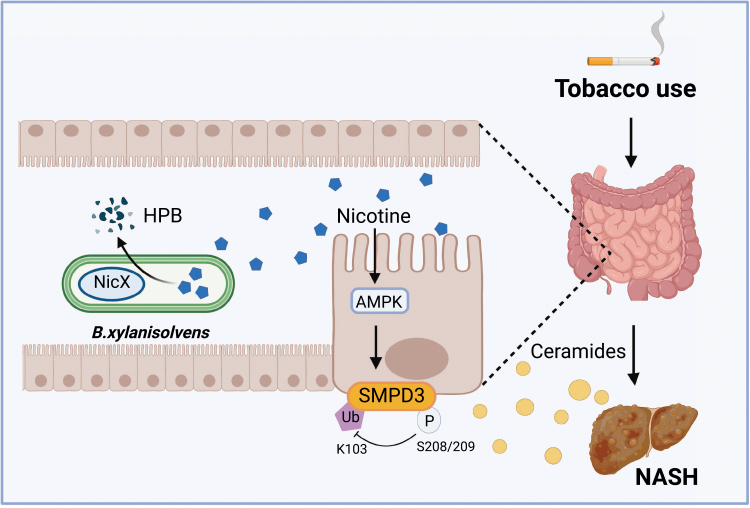
Tobacco use induces nicotine accumulation in the intestine and activates intestinal AMPKα-SMPD3-ceramide formation, contributing to NASH development. Gut bacterium *Bacteroides xylanisolvens*, as an effective nicotine degrader, can effectively reduce intestinal nicotine levels and delay the nicotine-exacerbated NAFLD progression.

The fact that smoking affects ulcerative colitis and Crohn’s disease suggests that the gut may be an important site for nicotine action. However, it is difficult to associate smoking with the gut due to the different ways that nicotine is administered, as well as the complexity of gut–nicotine interactions. Previous studies on nicotine mainly focused on its effects on organs such as the blood, lungs, and brain, although high levels of nicotine were also detected in saliva and gastric juice [10].

In this study, the research team quantitatively determined nicotine levels in terminal ileal mucosal biopsies, serum, and stool samples from 30 nonsmokers and 30 smokers. Surprisingly, they found very high nicotine levels in the ileal mucosal tissues of smokers. They also found similar results in ileum and ileal contents in different mouse models. These findings suggest that there is a huge accumulation of nicotine in the intestine as a result of smoking or nicotine exposure. Interestingly, nicotine concentrations in the ileum and its contents were higher in germ-free mice than those in specific-pathogen-free (SPF) mice after nicotine exposure, suggesting that the gut microbiota have the potential to degrade intestinal nicotine. Metagenomic analysis of smokers’ stool samples identified an intestinal bacterial species, *B. xylanisolvens*, which was the most significant bacterium correlated with intestinal nicotine levels. *In vitro* and *in vivo* experiments were conducted to verify the degradation of nicotine by *B. xylanisolvens*, results of which were further confirmed by the identification of nicotine degrading enzyme NicX and the metabolite 4-hydroxy-1-(3-pyridyl)-1-butanone (HPB). Nicotine treatment in mice exacerbated the progression of NAFL-NASH induced by a high-fat, high-fructose, high-cholesterol diet (HFHCD), whereas colonization with *B. xylanisolvens* alleviated the nicotine-promoted NASH progression in a NicX-dependent manner. These findings provide a novel therapeutic strategy for cigarette-associated NASH by targeting specific gut bacterium and their metabolic enzymes.

The team further found that nicotine accumulation in the intestine significantly activates AMPK signaling in intestinal epithelial cells. Compared with the structure of cotinine, the host metabolite of nicotine, HPB, the microbial metabolite of nicotine, showed a different structure that did not trigger AMPK activation. Combining phosphorylation proteomics and lipidomics approaches, the team revealed that nicotine activates phosphorylation at the S208 (S209 in humans) site of the ceramide-metabolizing enzyme sphingomyelin phosphodiesterase 3 (SMPD3) and promotes intestine-derived ceramide secretion. Mechanistic studies confirmed that SMPD3 is a novel phosphorylation substrate for AMPKα1. The team determined that phosphorylation at the S208 (S209) site could affect ubiquitination modification at the K103 site. By constructing mutant proteins and specific antibodies against the S208 (S209) site and the K103 site, they discovered that AMPKα1 could directly bind and phosphorylate the S208 (S209) site of SMPD3, thereby inhibiting the ubiquitination of the K103 site, which in turn ultimately increased SMPD3 activity by inhibiting the SMPD3 proteasomal degradation pathway. This important finding also establishes a new mechanistic link between AMPK signaling and sphingolipid metabolism.

Finally, the team conducted metagenomics and metabolomic analyses of the stool samples from 83 patients with liver biopsy-proven NAFLD, comprising 41 smokers and 42 nonsmokers. The results confirmed that nicotine levels in the smokers’ stools were positively associated with NASH progression, while *B. xylanisolvens* abundance and the nicotine-derived degradation product HPB were negatively associated with NASH progression. In contrast, there was no significant correlation between *B. xylanisolvens* abundance and NASH severity in the stools of the nonsmokers. These results suggest that *B. xylanisolvens*-mediated nicotine degradation exerts a significant protective effect against liver damage during NASH progression.

In summary, this study is an exciting and significant biomedical advance that improves our understanding of how nicotine in the gut of smokers promotes the development of NASH and how it is degraded by gut microbiota (Fig. 1). An essential aspect of this study is the translational potential that offers the hope of treating smoking-related NASH with beneficial bacteria. If we take a closer look at this novel nicotine degrader identified in the study, *B. xylanivsolvens* is free of toxin genes, *bft*, or *PSA*, unlike the enterotoxigenic bacterium, *Bacteroides fragilis*. Additionally, *B. xylanivsolvens* does not possess plasmid material in the genome and is not capable of colonizing in the intestines. All of these features show great promise of such a bacterium as a biologically safe probiotic option. Currently, heat-inactivated *B. xylanisolvens* has been supplemented to fermented food products such as yogurt as a probiotic in European countries. It is foreseeable that live probiotic *B. xylanisolvens* is going to be used in the future as a new treatment or adjuvant therapy for nicotine-induced NASH and perhaps, other smoking-related conditions as well.

There is a question, however, that still remains unanswered as to why smoking causes high concentrations of intestinal nicotine. One possible explanation is that digestive glands, especially intestinal glands, can retain nicotine, which ultimately leads to intestinal nicotine enrichment. There is also a good possibility that there is an enterohepatic circulation in the process of hepatic metabolism of nicotine, resulting in a high concentration of intestinal nicotine. Such an intriguing question certainly warrants further investigation in the future.
